# Initial HIV mucosal infection and dendritic cells

**DOI:** 10.1002/emmm.201202763

**Published:** 2013-05-07

**Authors:** Anthony L Cunningham, Andrew Harman, Najla Nasr

**Affiliations:** Centre for Virus research, Westmead Millennium Institute and University of SydneySydney, Australia

**Keywords:** anogenital and intestinal epithelia, CCR5, dendritic cells, HIV, viral transfer

HIV hijacks the body's immune system to have itself delivered to T cells in which it can explosively replicate. It does this partly by subverting the function of various types of dendritic cells (DCs) that are present in many of the tissues exposed to the external environment. To date, however, it has been unclear how HIV is transmitted in the gut. A recent paper in EMBO Molecular Medicine goes much of the way to elucidating this mechanism, implicating DCs in the lamina propria that sit beneath the DC-free columnar epithelia (CE) of the gut and extend and then return processes between the CE cells to capture HIV (Cavarelli et al, 2013).

Dendritic cells link innate and adaptive immune mechanisms, making them a vital cell type in our defence against pathogens. Their role is to capture pathogens, which they process and present their antigens to T cells. They are also in constant communication with other immune cells and each other. They protect surrounding cells from pathogens by secreting type I and III interferons (IFN), and promote the migration of other immune cells to the site of infection through the release of cytokines and chemokines. DCs are derived originally from bone marrow and divided into two major types: the myeloid and plasmacytoid DCs (which are major producers of type I interferons). Myeloid DCs consist of sub-populations found in diverse anatomical locations throughout the body, and especially in tissues in contact with the external environment. These include the blood, secondary lymphoid tissue and especially the anogenital and intestinal epithelium. DC sub-populations have been best defined in mice, but the human homologues are now being described (Ziegler-Heitbrock et al, 2010).

In mucosal stratified squamous epithelia (SSE), immature Langerhans cells (LC) – a type of DC – express the C-type lectin receptor (CLR) langerin and patrol this epithelium which covers the lower female genital tract, anus and male foreskin in humans. Myeloid DCs thus act as sentinels, specialized for pathogen detection. The underlying dermis contains at least three subsets of DCs that are characterized by their surface receptors: CD1a, CD14 or, as recently discovered, blood dendritic cell antigen 3 (BDCA3). Most express CLRs, a mannose receptor and/or DC-specific intercellular adhesion molecule-3-grabbing nonintegrin (DC-SIGN), as well as different complements of Toll-like receptors (TLRs). Unlike the SSE, the CE coating the respiratory tract, gut and endocervix, is devoid of resident DCs. However, DC populations found in the underlying lamina propria closely resemble dermal DCs (Cunningham et al, 2013; Harman et al, 2013).

DCs mature after encountering pathogens and presenting antigens to T cells, either in the mucosa, or after migration to lymph nodes. DCs thus represent an ideal vehicle to be hijacked by HIV to gain transport to T cells.

DCs thus represent an ideal vehicle to be hijacked by HIV to gain transport to T cells.

All DCs express the HIV entry receptors CD4 and CCR5 in their immature state. However, during maturation and migration, CCR5 is down-regulated and CXCR4 begins to be expressed. Thus, HIV may interact with DCs at various mucosal sites during sexual transmission and at intestinal mucosa during parturition. HIV targets LCs in SSE unless genital ulcers, such as those caused by Herpes simplex virus, are present: herpes is known to enhance HIV acquisition three- to fourfold. Although several reports that have used *ex vivo* human cervical and foreskin explants strongly suggest that LCs are initial targets in the genital mucosa, this has proven difficult to show *in vivo* (Hladik et al, 2007; Zhang et al, 1999; Zhou et al, 2011). It is still unknown whether DCs play a role in HIV uptake in intact CE, although the DCs in the underlying lamina propria are known to extend processes through to the epithelial surface. However, their interactions with HIV are still poorly understood.

Thus, the paper by Cavarelli et al. in this month's EMBO Molecular Medicine makes some important progress to answering this question (Cavarelli et al, 2013). The authors use human intestinal explants to elucidate mechanisms of HIV transfer across the CE of the colon. They clearly demonstrate that lamina propria DCs extend and return processes containing HIV between CE cells: a clear proof of principle of an HIV-DC uptake mechanism in the gut (Fig 1).

Lamina propria DCs extend and return processes containing HIV between CE cells: a clear proof of principle of an HIV-DC uptake mechanism in the gut.

It is also relevant to infection after anal intercourse, where semen is delivered above the anal valve where the mucosa transitions from SSE to CE.

**Figure 1:**
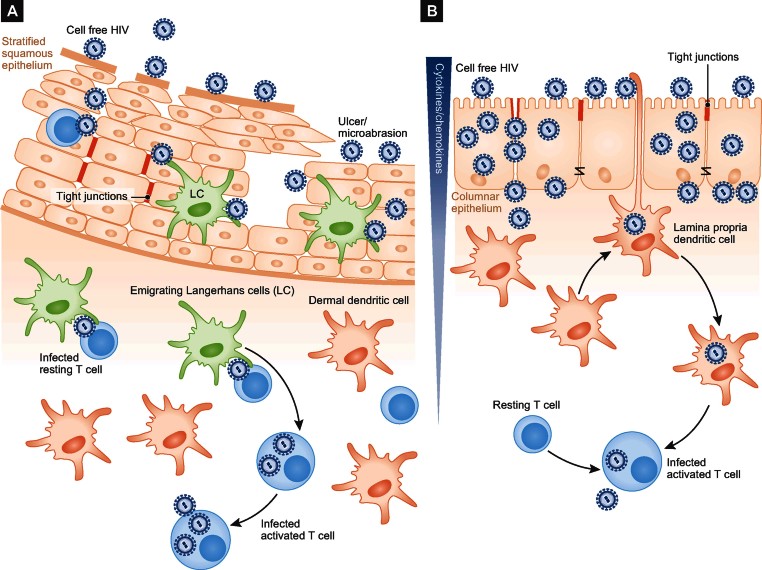
Role of DCs in the penetration of anogenital and intestinal epithelial surfaces by HIV. A. Penetration through the stratified squamous epithelium. HIV comes into contact with Langerhans cells (LC) either by passing between keratinocytes or directly in the presence micro-abrasions or genital ulcers caused by sexually transmitted infections. LCs then migrate to the submucosa or lymph nodes transferring HIV via viral synapses to mostly resting T cells. B. Penetration through the columnar epithelium. HIV initially penetrates the tight junctions between columnar epithelial cells, establishing a viral concentration gradient that induces extension of processes from lamina propria DCs (or emigration of whole DCs), via an interaction with CCR5. Captured and/or replicating HIV is then transferred to activated T cells in the lamina propria. Alternatively, HIV may cross the columnar epithelium by transcytosis.

Many pathogens utilize DCs for transport and transfer to target cells, as they are thus protected from antibodies. In doing so, they manipulate the DC intracellular environment leading to loss, gain or alteration of DC functions. HIV is particularly adept at this. After binding to CLRs, most of the virus is taken up into neutral pH tetraspanin expressing vesicular caves, but a minor portion (<10%) is transferred to CD4/CCR5 receptors, leading to neutral fusion with the plasma membrane. Once the infected DC contacts CD4 T cells, the large portion of HIV residing in the vesicular caves is transferred to the T cells by expulsion into the virologic synapse at the contact region. HIV also enters the endosomal system where it is lysed by acid-proteolytic digestion prior to antigen presentation to T cells. HIV entering via the CD4/CCR5 receptor undergoes *de novo* replication followed by process formation with HIV at the tip. The tip makes contact with the T cells and transfers the virus to them at a second, later stage of transfer. HIV uptake induces two corresponding phases of DC gene expression, many of which facilitate viral transfer and replication: Type I and III IFN expression is inhibited at early and late stages of infection; DCs and LCs undergo enhanced maturation and migration (via CCR7); adhesion molecules are up-regulated (ICAM1) and lysosomal enzyme expression is down-regulated. The viral and cellular stimuli modulating such changes are still being elucidated. However, we do know that the HIV gene and protein product Vpr inhibits type I IFN production, and that signalling through CCR5 and components of the HIV inoculum might induce maturation and migration. HIV nucleic acids might interact with cytoplasmic RNA or DNA sensors to induce these changes (Anand et al, 2009; Cunningham et al, 2013; Harman et al, 2011).

The paper by Cavarelli et al. extends our knowledge of such mechanisms. They have shown that HIV exposure to the intestinal mucosa allows viral penetration through transiently open tight junctions of the CE, thus creating a gradient that attracts the emigration of lamina propria DCs and extension of their processes through this route.

HIV exposure to the intestinal mucosa allows viral penetration through transiently open tight junctions of the CE, thus creating a gradient that attracts the emigration of lamina propria DCs and extension of their processes through this route.

This response was dependent upon the interaction of the HIV envelope protein with CCR5, but not CXCR4. This viral protein is known to signal through CCR5 and to induce DC migration (Anand et al, 2009; Zhou et al, 2011). HIV, using either CXCR4 or CCR5, can also be transcytosed across epithelial cells to be captured by DCs. Although lamina propria DC processes have been shown to extend into the intestinal lumen constitutively or after HIV exposure, this does not seem to occur with LCs in SSE. The reasons for these differences, and also whether transfer of HIV to activated T cells occurs via *de novo* replication or vesicular caves, will be interesting to elucidate.

These studies provide a further rationale for developing inhibitors of HIV-CCR5 interactions as microbicides, especially in the anal mucosa and perhaps in combination with suppressive therapy in late pregnancy and delivery (Lederman & Este, 2009). However, it also indicates the need for more knowledge about the pathogenesis of anorectal infection: how often does the virus enter via the columnar epithelium above the anal valves? Macrophages in the anal mucosa have recently been shown to express CCR5, unlike those in the intestinal mucosa. Is it possible that anal mucosal macrophages are also important in transmission? Furthermore, does the interaction of HIV with CCR5 on LCs in the SSE result in similar (but more limited) migration that is relevant to viral entry?

The authors declare that they have no conflict of interest.

## References

[b1] Anand AR, Prasad A, Bradley RR, Deol YS, Nagaraja T, Ren X, Terwilliger EF, Ganju RK (2009). HIV-1 gp120-induced migration of dendritic cells is regulated by a novel kinase cascade involving Pyk2, p38 MAP kinase, and LSP1. Blood.

[b2] Cavarelli M, Foglieni C, Rescigno M, Scarlatti G (2013). R5 HIV-1 envelope attracts dendritic cells to cross the human intestinal epithelium and sample luminal virions via engagement of the CCR5. EMBO Mol Med.

[b3] Cunningham AL, Harman A, Kim M, Nasr N, Lai J (2013). Immunobiology of dendritic cells and the influence of HIV infection. Adv Exp Med Biol.

[b4] Harman AN, Bye CR, Nasr N, Sandgren KJ, Kim M, Mercier SK, Botting RA, Lewin SR, Cunningham AL, Cameron PU (2013). Identification of lineage relationships and novel markers of blood and skin human dendritic cells. J Immunol.

[b5] Harman AN, Lai J, Turville S, Samarajiwa S, Gray L, Marsden V, Mercier SK, Jones K, Nasr N, Rustagi A (2011). HIV infection of dendritic cells subverts the IFN induction pathway via IRF-1 and inhibits type 1 IFN production. Blood.

[b6] Hladik F, Sakchalathorn P, Ballweber L, Lentz G, Fialkow M, Eschenbach D, McElrath MJ (2007). Initial events in establishing vaginal entry and infection by human immunodeficiency virus type-1. Immunity.

[b7] Lederman MM, Este J (2009). Targeting a host element as a strategy to block HIV replication: is it nice to fool with Mother Nature. Curr Opin HIV AIDS.

[b8] Zhang Z, Schuler T, Zupancic M, Wietgrefe S, Staskus KA, Reimann KA, Reinhart TA, Rogan M, Cavert W, Miller CJ (1999). Sexual transmission and propagation of SIV and HIV in resting and activated CD4+ T cells. Science.

[b9] Zhou Z, Barry de Longchamps N, Schmitt A, Zerbib M, Vacher-Lavenu MC, Bomsel M, Ganor Y (2011). HIV-1 efficient entry in inner foreskin is mediated by elevated CCL5/RANTES that recruits T cells and fuels conjugate formation with Langerhans cells. PLoS Pathogens.

[b10] Ziegler-Heitbrock L, Ancuta P, Crowe S, Dalod M, Grau V, Hart DN, Leenen PJ, Liu YJ, MacPherson G, Randolph GJ (2010). Nomenclature of monocytes and dendritic cells in blood. Blood.

