# Corrigendum

**DOI:** 10.1002/emmm.201370102

**Published:** 2013-10-02

**Corrigendum to** Anthony L. Cunningham, Andrew N. Harman, Najla Nasr (2013) Initial HIV mucosal infection and dendritic cells. EMBO Mol Med 5 (658–660) DOI: 10.1002/emmm.201202763

The name of the second author in this article, “Andrew N. Harman,” missed the middle initial N. The correct author names are listed above.

